# Engineering of III-Nitride Semiconductors on Low Temperature Co-fired Ceramics

**DOI:** 10.1038/s41598-018-25416-6

**Published:** 2018-05-02

**Authors:** J. M. Mánuel, J. J. Jiménez, F. M. Morales, B. Lacroix, A. J. Santos, R. García, E. Blanco, M. Domínguez, M. Ramírez, A. M. Beltrán, D. Alexandrov, J. Tot, R. Dubreuil, V. Videkov, S. Andreev, B. Tzaneva, H. Bartsch, J. Breiling, J. Pezoldt, M. Fischer, J. Müller

**Affiliations:** 1 IMEYMAT: Institute of Research on Electron Microscopy and Materials of the University of Cádiz, Cádiz, Spain; 2 Department of Condensed Matter Physics, Faculty of Sciences, University of Cádiz, Cádiz, Spain; 3 Department of Materials Science and Metallurgic Engineering, and Inorganic Chemistry, Faculty of Sciences, University of Cádiz, Cádiz, Spain; 4 Instituto de Ciencia de Materiales de Sevilla (CSIC-Universidad de Sevilla), Seville, Spain; 5 Department of Electrical Engineering, Lakehead University, Ontario, Canada; 6 Microelectronics Department, Technical University of Sofia, Sofia, Bulgaria; 7 Department of Chemistry, Technical University of Sofia, Sofia, Bulgaria; 8 Institut für Mikro- und Nanotechnologien MacroNano®, Technische Universität Ilmenau, Ilmenau, Germany; 9 Present Address: Dpto Ingeniería y Ciencia de los Materiales y del Transporte, Univ. de Sevilla, Seville, Spain

## Abstract

This work presents results in the field of advanced substrate solutions in order to achieve high crystalline quality group-III nitrides based heterostructures for high frequency and power devices or for sensor applications. With that objective, Low Temperature Co-fired Ceramics has been used, as a non-crystalline substrate. Structures like these have never been developed before, and for economic reasons will represent a groundbreaking material in these fields of Electronic. In this sense, the report presents the characterization through various techniques of three series of specimens where GaN was deposited on this ceramic composite, using different buffer layers, and a singular metal-organic chemical vapor deposition related technique for low temperature deposition. Other single crystalline ceramic-based templates were also utilized as substrate materials, for comparison purposes.

## Introduction

Group-III nitrides (III-N), especially those related to GaN and AlN, have been strategic semiconducting materials in Power Electronics (PE) for the last 30 years, due to their outstanding temperature stability and dielectric strength combined with a wide bandgap^1^. However, up to date, large volumes of these materials have not been obtained, and, therefore, bulk III-N substrates with extensive surfaces are not available yet. Traditionally the chosen templates for depositions of nitrides are wafers of sapphire, silicon and silicon carbide. These substrates, however, imply disadvantages related to either a high dielectric constant, high leakage currents or to high economic costs.

Nevertheless, it still should be possible to lower the production prices by using composite materials made of ceramic fillers embedded into a glass matrix during a sinter process as a base, which demonstrated to be suitable for high-frequency circuits^2^. Among manifold advantages, the use of low temperature co-fired ceramics (LTCC) as an alternative glass-ceramic substrate enables tailoring of crucial properties, such as permittivity or coefficient of thermal expansion (CTE), thus enhancing the systems figures of merit. It can be argued, though, that this material is not fit for PE applications due to its low thermal conductivity, 2-5 W/mK^3^, but this issue can be overcome by placing metallic via arrays under the high power device^4^, which is feasible in the LTCC technology.

In principle, the production of LTCC materials is not cheaper than that of Si itself, but the fact that it can be processed layer by layer while in green state eases the fabrication, by using masks and drills, of inner channels for the allocation of a metallic network acting as electrical connectors, passive circuits and thermal drains. Since this architecture of contacts can be placed prior to the production of the active layers of the devices, this leads to a further reduction of both costs and size, as well as to an improvement of the whole assembly performance^5^. Nevertheless, two main handicaps impede the implementation of III-N growth technologies on LTCC-based substrates by often needed conditions: (i) the inherent, relatively high roughness and porosity of this support lead to low homogeneities in III-N structure and composition; (ii) the often used III-N growth temperatures (over 700 °C) may promote ceramic damage and metal contamination. These are the main reasons why the potential of III-N-on-LTCC has still not been fully exploited in order to obtain the atomically long-range periodic structures that are needed for PE active devices, such as High Electron Mobility Transistors (HEMTs), although the possibilities of a LTCC hybrid technology for passive circuit components in such field have already been explored for over a decade^6–10^.

In this work, we show the results after the first stages of novel proposals to overcome the challenges in III-N/LTCC production and, therefore, the first steps in the production of a revolutionary material for power and high-frequency electronics. In this way, worthy qualities of GaN epilayers were reached by: (i) new recipes for improving surface flatness, chemical affinity and CTE matching between LTCC and III-Ns; (ii) the use of intermediate preparation or buffer layers; (iii) the choice of techniques for the synthesis of III-N crystal layers at temperatures much lower than conventional. The GaN quality progressive improvement, up to the achievement of the best quality so far obtained, to the best of our knowledge, for a III-N layer grown on a LTCC (and similar ceramics) substrate, is reported.

## Results and Discussion

Different aspects of the fabricated materials are discussed in this section: surface roughness, layers architecture, topology, chemical composition, and crystallographic structure. A comprehensive view of the studied sets of samples is presented in Table 1. The average thickness values in this table have been obtained through low magnification conventional electron microscopy, in the case of the thickest layers; and using atomically resolved electron micrographs, for the thinnest ones. The error bars correspond, in each case, to the statistical error of the measurements, taking into account the microscope maximum resolution and the pixel size of the images.

**Table 1:** Sample nomenclature in this work, layers sequence and their average thicknesses, obtained from transmission electron microscopy, and GaN growth temperature.

Label	Substrate	Finishing	Leveling layer		AlN layer thickness (nm)	GaN growth temperature (°)	GaN layer thickness (nm)
			Type	Thickness (nm)			
CT1	CT 700	as fired	Al_2_O_3_	2600 ± 10	53 ± 5	550	25.9 ± 2.2
CT2	CT 700	as fired	sol gel	1200 ± 10	30.1 ± 2.1	550	14.9 ± 1.2
CT3	CT 700	polished	Al_2_O_3_	490 ± 40	84.26 ± 10	540	59 ± 4
CT4	CT 700	polished	Al_2_O_3_	250 ± 30	—	550	518 ± 22
ST1	Sitall	polished	Al_2_O_3_	300 ± 70	48 ± 4	540	21.0 ± 0.2
ST2	Sitall	polished	Al_2_O_3_	439 ± 15	—	550	605 ± 30
SC1	SiCer (111)	polished	SiO_2_	2.0 ± 0.2	—	550	565 ± 9
SC2	SiCer (100)	polished	SiO_2_	2.0 ± 0.5	—	550	590 ± 14

### Surface roughness and layers architecture

#### Surface Roughness

Some materials utilized as bulk substrates for the nitride-based heterostructures in this work are of ceramic nature, therefore, surface roughness and porosity are, as previously mentioned, very important issues regarding the achievement of a good crystalline structure for the top layers. If this substrate roughness is not low enough, the first depositions of Ga and N atoms will most probably be inhomogeneous, promoting regions of different crystallographic orientations. Therefore, following the fabrication of the LTCC substrates through a sintering process^11^, a well-established polishing/lapping^12^ method was applied to LTCC substrates in samples CT3 and CT4 (not in samples CT1 or CT2) before proceeding to further element deposition. A 20-minutes lapping was applied using B_4_C as abrasive material followed by a 90-minutes polishing step with 1 µm-size diamond particles. By means of this methodology, the achieved LTCC surface roughness (taking into account the pores) had a root mean square deviation (RMS)^13^ of *R*_q_ = (120 ± 5) nm.

**Figure 1:**
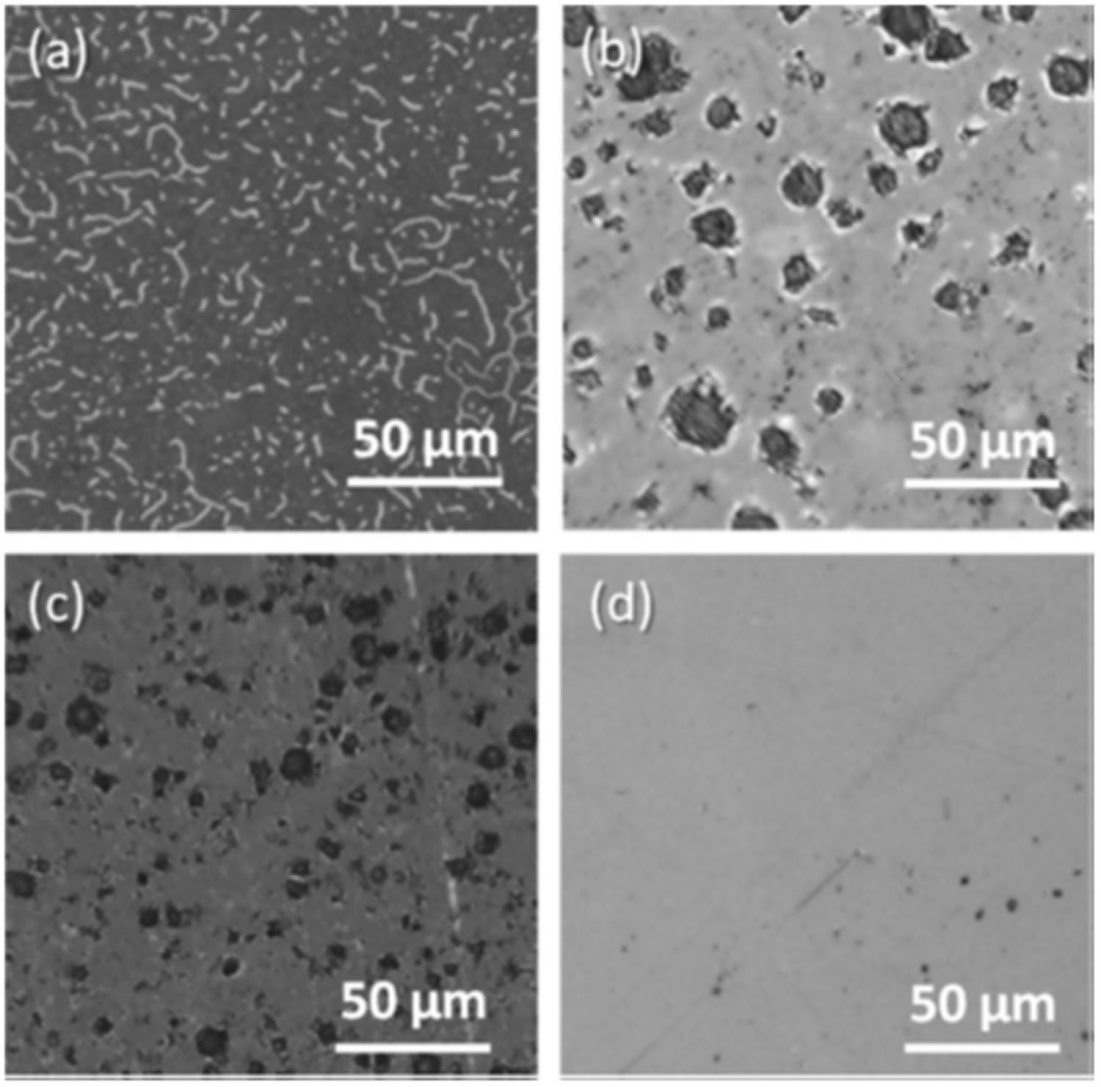
Images of surfaces of materials from samples CT1. (**a**) CT3 (**b**) CT4 (**c**) and ST2 (**d**) obtained with an optical microscope.

The LTCC surface is closed after firing, so the surface roughness is determined by the powder fraction of the ceramic filler (crystalline) contained into the glass (amorphous). Moreover, the polishing opens the intrinsic pores of the material, which are always present. Size and density of these pores depend on the powder composition of the tape and the sintering conditions. Though the so achieved LTCC surfaces are smoother than their non-treated counterparts, the substrate topography still led to the formation of pits in the material surface because occasionally larger pores are opened by the detachment of the filler particles, promoting a rough surface along the overgrown GaN at these positions. Besides, the LTCC roughness limits the deposition rate and the crystallinity of the nitride, which impedes to continue growing a thicker GaN layer as a roughness relieving solution to this problem. As a first step in the characterization of the materials, the amount and density of pits and surface features for the GaN was observed by optical microscopy, as shown in Fig. 1. The first set (samples CT1 and CT2) presented microcracks-like features along its surface, as it can be observed in Fig. 1(a), being one of the main explanations for this, the fact that the LTCC substrate was not previously polished. On the other hand, samples CT3 and CT4 (Fig. 1 (b)–(c)), for which the substrate grinding process promoted the formation of open surface pores, localized pits were the main issue regarding the GaN surface roughness. This did not happen with structures using SiCer and Sitall (Fig. 1(d)), where no pores are expected in the substrate. Note that the brightness of the microcracks in Fig. 1(a) is produced by the reflecting light utilized to illuminate the material in the optical microscope, while the pores, deeper and broader features, reflect no light and, therefore, appear as dark spots in the other images in Fig. 1.

**Figure 2:**
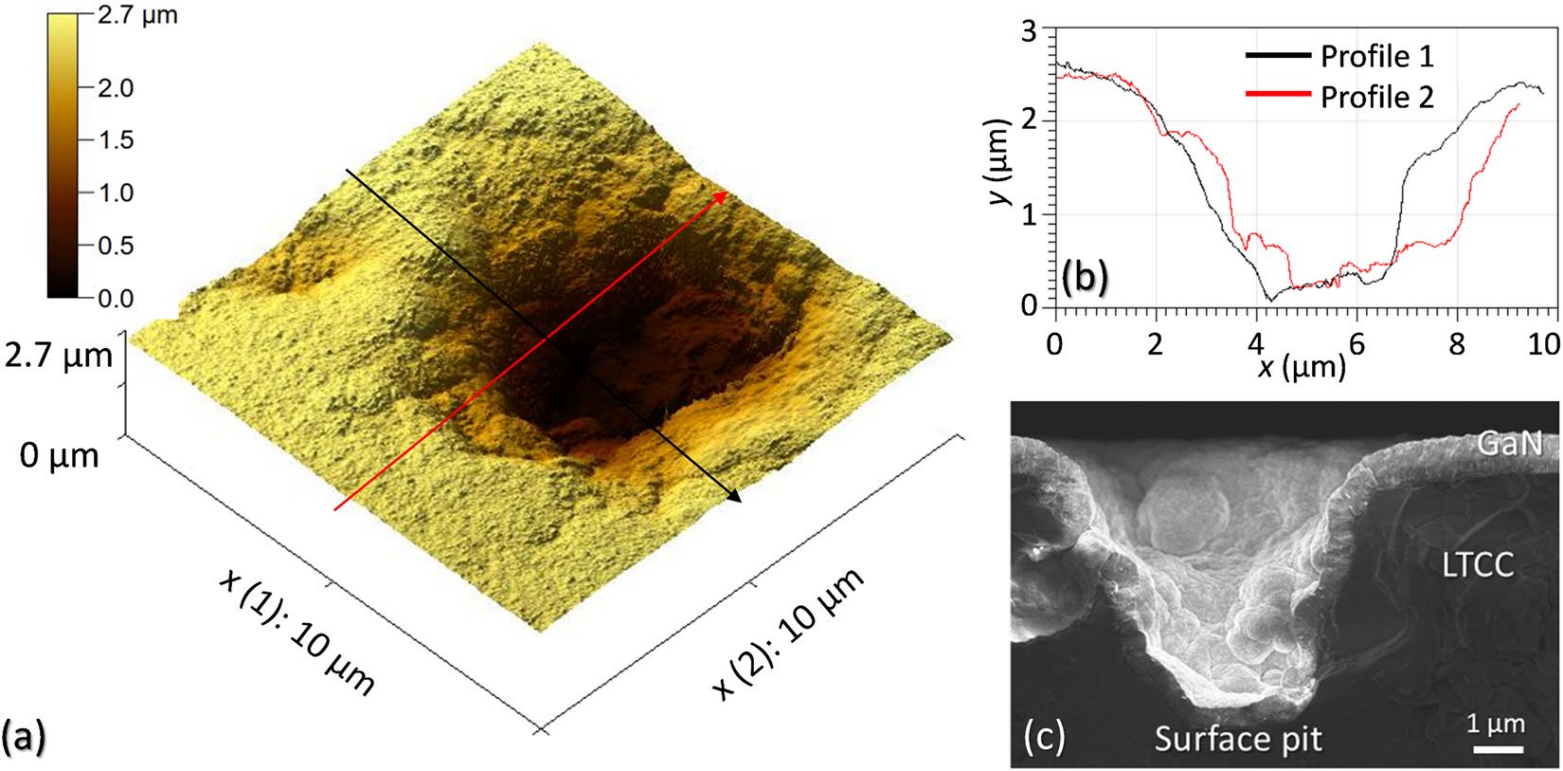
Surface pits in sample CT4: AFM 3D simulation (**a**) with two lines indicating the perpendicular directions of depth profiles across the pit (**b**) and SEM micrograph of cross section for another pit (**c**).

In addition to this, other techniques, such as Atomic Force and Electron Microscopies, were used at the University of Cádiz (UCA) in order to obtain a more detailed view of these pits, specifically regarding to their depths. This technique was applied by using NT-SS-I SuperSharp tips from Next-Tip SL (with an approximate nominal radius of 2 nm) to obtain Atomic Force Microscopy (AFM) images, which were afterwards processed with the Gwyddion software^14^. Figure 2(a) shows a three dimensional simulation obtained from AFM intensity images of a pit in the surface of sample CT4. Two perpendicular directions have been selected to obtain depth profiles of this pit (Fig. 2(b)). This pit is representative also for those in sample CT3, with a typical depth of about 3 µm. Such structures are expected when used grinded/polished CT 700, as previously commented. Also in this image, a Scanning Electron Microscopy (SEM) micrograph reveals a cross-section of a similar pit in the same specimen. It can be observed how the GaN fills partially the pit, but due to its profoundness, it is not possible for the nitride to grow in a 2D-mode or not even to completely fill the hole.

**Figure 3:**
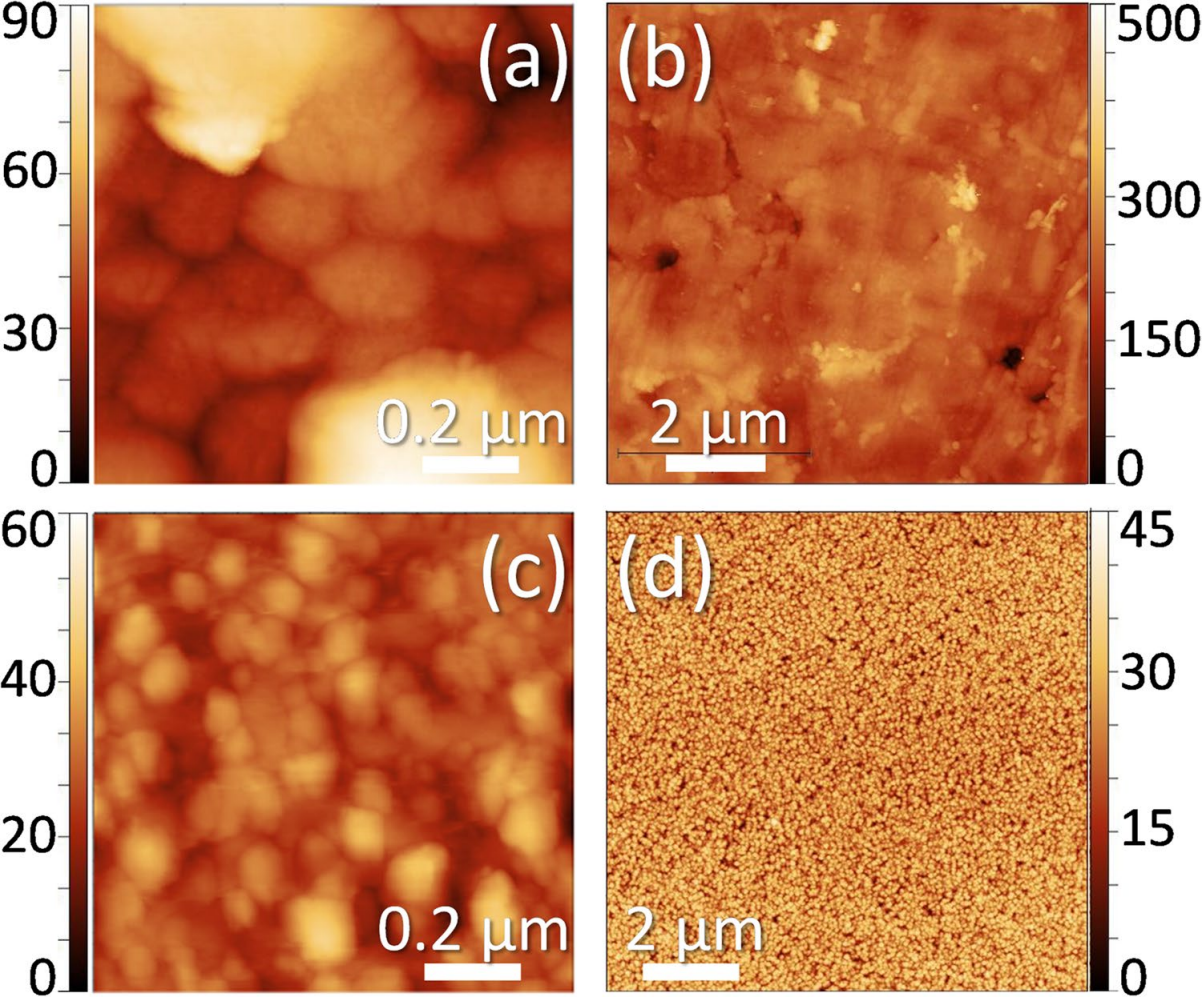
AFM intensity maps for surfaces of samples CT3. (**a**) ST1 (**b**) CT4 (**c**) and SC1 (**d**). The color-scales for the depth are given in nanometers.

On a more general application, AFM was used to quantify the roughness of the GaN surfaces. Representative areas of such surfaces (between pores) are shown in Fig. 3, while Table 2 collects AFM obtained values for RMS surface roughness (*S*_q_)^13^. It has to be taken into account that the values in this table indicate pore to pore measurements, using 1 × 1 µm^2^ areas. Note that if those areas included the surface pits, which could be the case of carrying out the calculations using AFM maps for 5 × 5 or 10 × 10 µm^2^ areas, S_q_ values would increase enormously for the samples using LTCC, while they would not significantly change for those samples using non-porous substrates. For example, when studying a 10 × 10 µm^2^ AFM maps, S_q_ values for the GaN surface in samples CT4, ST1, SC1 and SC2 turn out to be 672, 30.0, 5.0 and 7.4 nm, respectively. This is a clear indication than the porosity in GaN layer is mainly produced by the porosity in the substrate. In any case, a clear evolution is observed regarding the decrease of the surface roughness of GaN structures along the successive set of samples and growth processes, being remarkable the fact that the roughness values in samples using LTCC, when the growth process is modified, became close to the ones in heterostructures using other substrates with different growth processes.

**Table 2:** Surface roughness (RMS) values for GaN surfaces for the different materials, obtained from AFM. The error values have been obtained through the statistical treatment of three different S_q_ measures for each sample (NM stands for “not measured”).

Sample	CT1	CT2	CT3	CT4	ST1	ST2	SC1	SC2
S_q_ (nm)	95.7 ± NM	26.74 ± 5.1	17.8 ± 0.4	5.5 ± NM	11.9 ± 2.6	10.2 ± 1.8	5.4 ± 0.3	4.8 ± 0.6

#### Layers architecture

**Figure 4:**
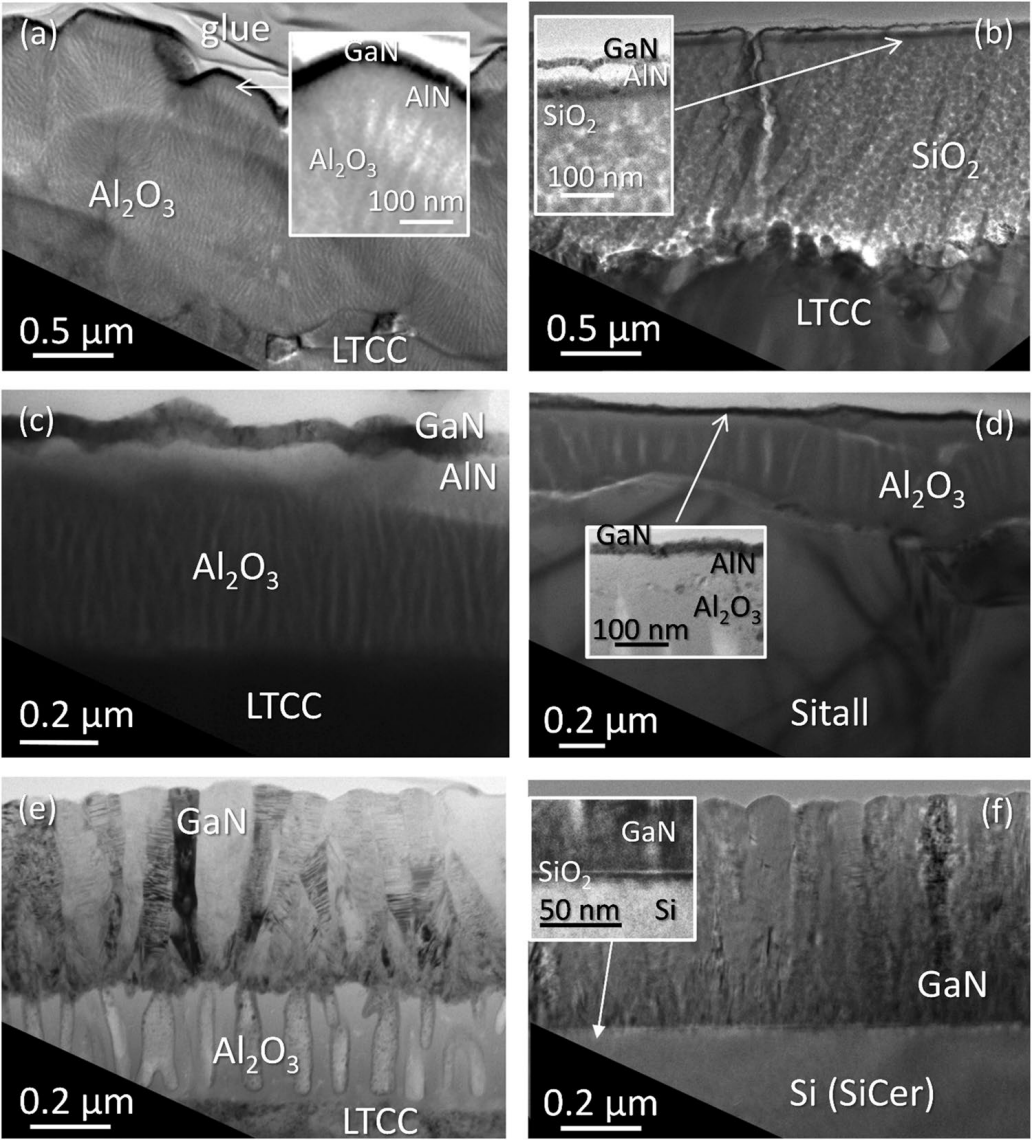
BF-DCTEM micrographs for XTEM preparations of samples CT1. (**a**) CT2 (**b**) CT3 (**c**) ST1 (**d**), CT4 (**e**) and SC1 (**f**).

Although all heterostructures present a GaN top layer, the layer thicknesses and stacking sequence differ significantly from one specimen to another, also within the same series. The thicknesses, summarized in Table 1, were obtained by Transmission Electron Microscopy (TEM) in Conventional and High Resolution modes (CTEM, HRTEM), which also gave useful information on the micro- and nanostructure of those materials. Figure 4 reveals this information for selected samples using different bulk substrates, intermediate and buffer layers, for comparison purposes. The micrographs in this figure were obtained in Bright Field Diffraction Contrast TEM (BF-DCTEM) mode, orienting the cross-section (XTEM) preparations parallel to the electron beam in the column of the TEM microscope, so the detected thicknesses for each layer were the maximum possible.

At a first glance, it is clear that the sample using unpolished CT 700 substrate and Al_2_O_3_ as an intermediate layer (CT1) has the most irregular surface formation, since this Al_2_O_3_ is achieved as a conglomerate of non-intentional conic-like features, on top of which the AlN/GaN sequence was grown. Surprisingly enough, the layers on top of the Al_2_O_3_ were continuous and quite homogeneous along the whole surface. Such BF-DCTEM images explain the look of optical micrographs in Fig. 1(a) showing extended defects, which are originated by this uneven alumina formation. On opposition to this, the use of a SiO_2_ intermediate layer (sample CT2) generates a more uniform support for the AlN/GaN formation. This is clearly because the used sol-gel silica deposition, with a great capacity for filling cavities and surface planarization, smoothens the surface and suppresses the propagation of the LTCC roughness to upper layers. However, the SiO_2_ placement approach was not applied to substrate materials in other series, since despite that structural advantage, its CTE value is not adjusted to that of the LTCC, as it happens in the case of the Al_2_O_3_. Therefore, the use of an alumina interlayer was considered of more interest in the following series.

As previously mentioned, the heterostructures fabricated using RF-plasma (CT1-3 and ST1) make use of an AlN buffer layer, in order to obtain a better adjustment between the gallium nitride compound and the support below. Note that AlN was considered the best material for that purpose, due to a high chemical affinity to both underneath alumina and above gallium nitride, a more similar basal lattice parameter to GaN than other templates, and the promotion of two dimensional GaN nucleation. In fact, this material has been employed for more than 30 years as the most suitable buffer layer for the later growth of GaN^15,16^.

The intensity in BF-DCTEM micrographs of an electron-transparent material at certain regions decreases with the atomic density of the structure in the direction of the interacting electron beam. Therefore, the dark, thin GaN shown in the Fig. 4(a)–(d) suggest a polycrystalline nature. This assumption is supported by HRTEM results, discussed in further sections of the text. As a consequence of this reasoning about DCTEM images intensities, it can be understood that intensity contrasts in such micrographs indicate a change in the atomic plane orientation between two regions inside a crystalline structure. Thus, Fig. 4(e) and (f) show that the thick nitride layers in samples CT4 and SC1 are formed by vertical, relatively large crystalline domains, randomly tilted and slightly twisted one from another. The same is applicable to samples ST2 and SC2, though their corresponding images are not shown here. Therefore, TEM micrographs allow to conclude that the change in the GaN growth conditions, from Radio Frequency (RF) to Direct Current (DC) plasma source and its application during a longer period of time, led to a clear improvement in the crystallinity and thickness of the GaN along the sets of samples using LTCC substrates (CT1 to CT4), even achieving similar results than those with less porous substrates (ST1, ST2, SC1 and SC2).

**Figure 5:**
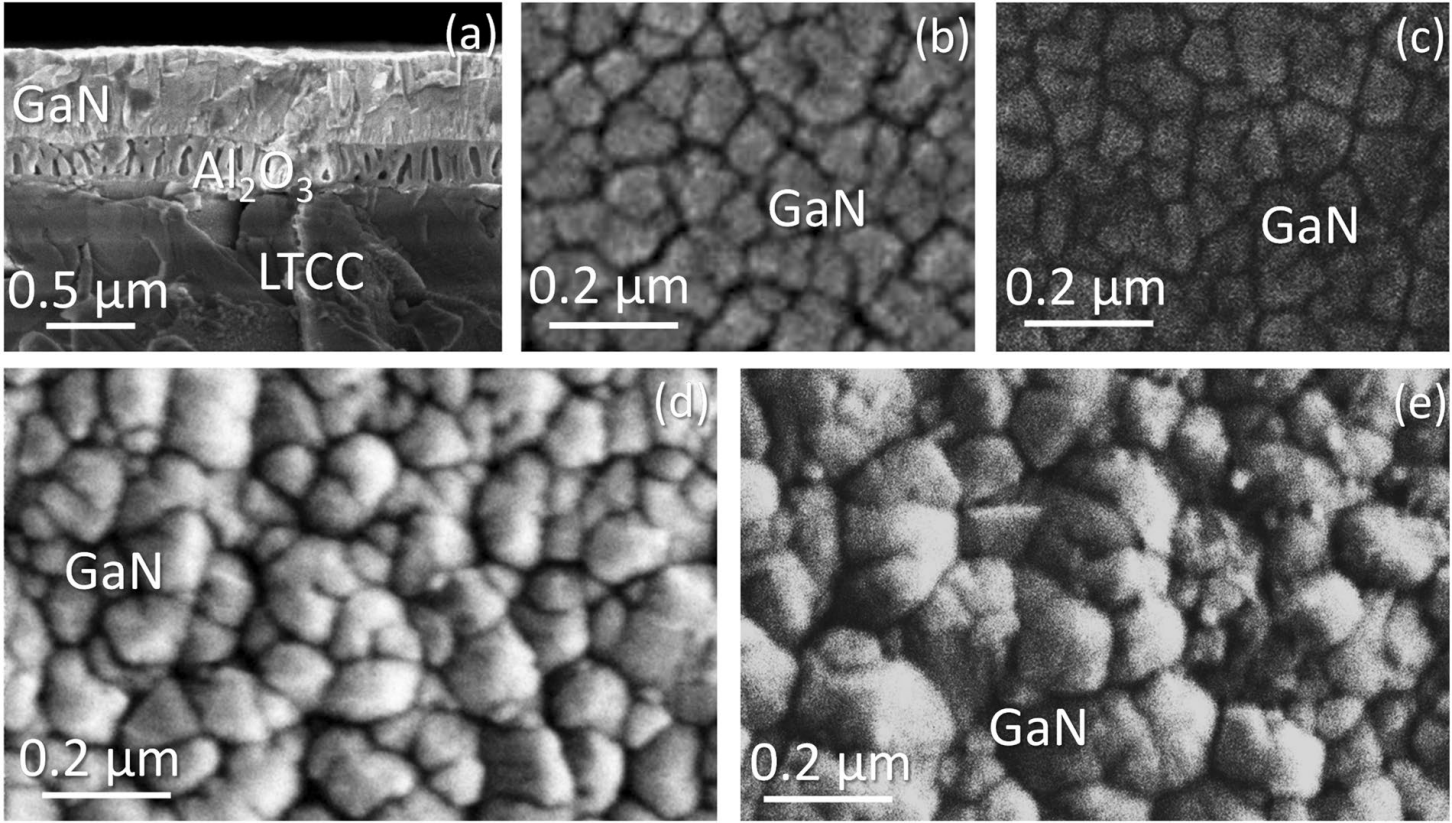
SEM cross section micrograph for sample CT4. (**a**) and planar view micrographs for samples SC1 (**b**) SC2 (**c**) ST2(**d**) and CT4 (**e**).

SEM micrographs in Fig. 5, for cross-section and planar (top) views, are in agreement with the previous affirmation, and show how the achieved thick GaN is distributed forming rotated crystallographic domains, in a so-called “mosaic microstructure”^17^. This structure is closer to a single crystal than those in the previous series, which are pure polycrystals. Clearly, the random orientation of those domains stems from the early stages of the growth. The roughness of this surface is still high enough to avoid a proper matching between the III-N structures during the coalescence process, and a multitude of grains remain differently oriented even along the whole growth process.

**Figure 6:**
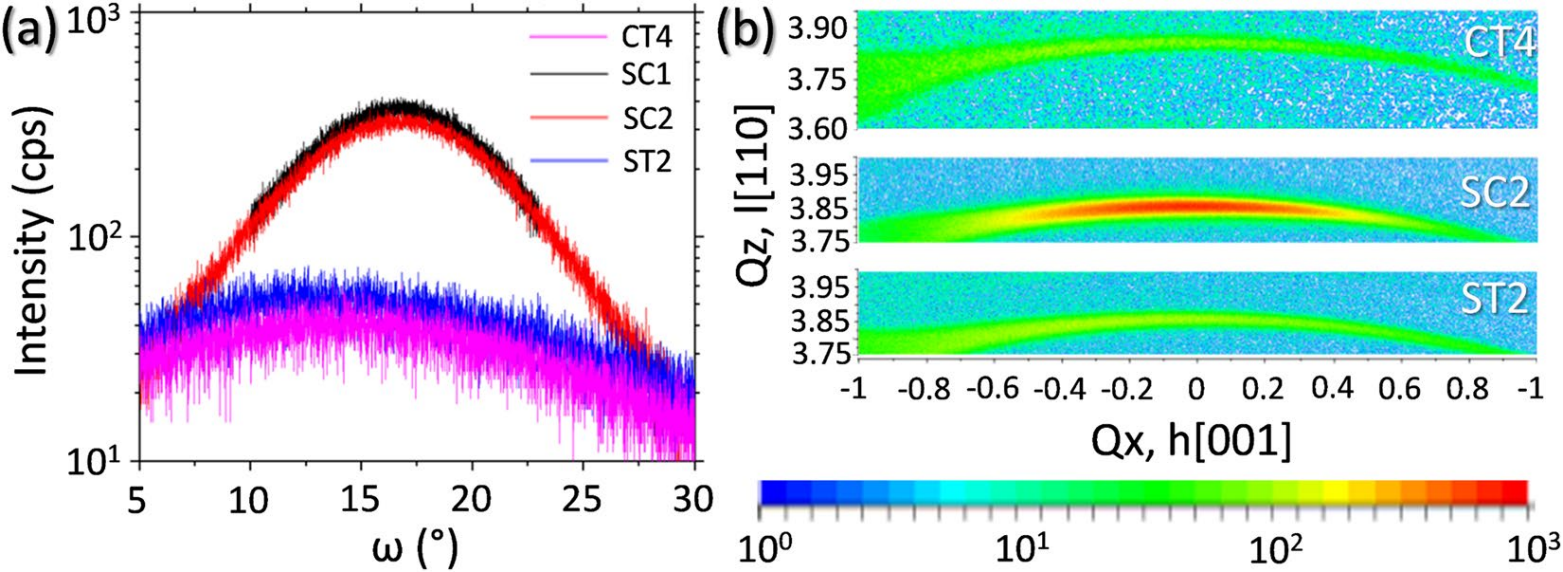
XRD rocking curves (**a**) and RSMs with color scale (**b**) for GaN(0002) peak in samples with mosaicity.

X-ray diffraction (XRD) was utilized to study the details of the mosaic structures on different substrate materials. Figure 6 shows rocking curves (omega scans) and reciprocal space maps (RSM) for the samples that presented these arrangements. Layers exhibiting defect free, low stressed, single crystalline GaN structures would produce very sharp peaks in omega scans, and highly symmetric ones in both kinds of XRD diffractograms. Nevertheless, it can be observed that this is not the case. Due to the disruption in the parallelism of atomic planes produced by defects such as mosaicity, the peaks are broadened and asymmetric, and RSM diffraction features show an arched shape, characteristic of this type of random arrangements.

### Topology

In the case of sample CT4, TEM images also reveal that the utilized Al_2_O_3_ is not a totally compact film, but presents columnar shapes with embedded elongated voids, as a result of an applied anodic treatment to the aluminum precursor of these layers (Fig. 4(e)). Nevertheless, due to TEM measurements local character, a logical question is whether or not this kind of structure formation can be observed across the substrate, i.e., what the process homogeneity is. Spectroscopic Ellipsometry (SE) is an ideal tool to answer that question since it can produce topographic maps of internal layers for macroscopic areas with a high thickness sensitivity (~0.1 Å)^18^. Besides, exclusively for this series, SE made with a 200 μm diameter probe from UV to NIR, fitted with a good mean square error to Cauchy functions for both GaN and Al_2_O_3_ layers on the 450–950 nm wavelength range, according to the equation $n=A+B/{\lambda }^{2}+C/{\lambda }^{4}+\cdots$.

The optical model used for the analysis of ellipsometric measurements was inspired in the SEM and HRTEM results and includes a multilayer structure of surface roughness/GaN/Intermix/Al_2_O_3_-Void/LTCC substrate. The roughness of the surface, which can be observed in Fig. 5(a), can be modeled as a layer which optical constants are calculated from the GaN layers by applying the Bruggeman effective medium approximation with a 50% of voids. On the other hand, the bonding zone between the alumina and the GaN, which as observed in the TEM image is not abrupt, is considered as an intermix layer in the model. This modeled layer represents a region in which its optical properties are 50% of Al_2_O_3_ and 50% of GaN contributions. Likewise, the non-uniformity of the thickness has been determined from the adjustment of the depolarization obtained during the SE experiment.

The best-fit thickness values for sample CT4, shown in Table 3, are in a very good agreement with those obtained from electron microscopy images (Table 1 and Figs 4(e) and 5(a)). Thus, a non-uniformity of a 3.5% would mean changes of around 18 nm in the GaN layer thickness along an area of 200 μm (the spot size of the SE probe).

**Table 3:** Best-fit thickness (in nm) values for the different layers and non-uniformity (in %) of GaN in sample CT4 obtained from SE.

Layer	Thickness (nm)
Roughness	11.3 ± 0.6
GaN	505.9 ± 1.6
Intermix	27.5 ± 2.3
Al_2_O_3_	299.8 ± 1.6
Non-uniformity (%)	3.52 ± 0.19

**Figure 7:**
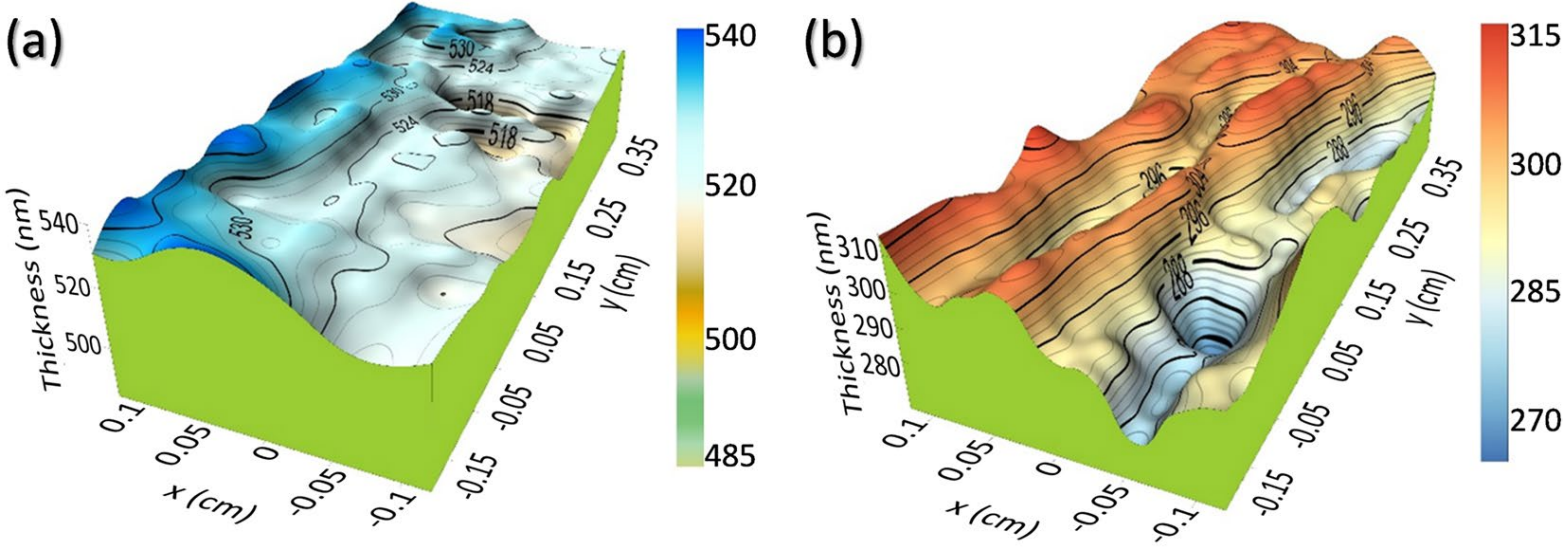
Topological maps for GaN (**a**) and Al_2_O_3_ (**b**) in sample CT4, obtained by SE.

The developed and verified optical model was applied to carry out thickness mapping investigations of buried and surface layers for several millimeter-sized regions. The maps were performed by SE scanning using the same 200 µm probe and the model of the Cauchy dispersion function previously applied. Figure 7 shows the topology maps obtained as a result of the measurements, both for the GaN surface layer (Fig. 7(a)), which is shown without the optical roughness, and for the Al_2_O_3_ layer below (Fig. 7(b)). It is evident that the GaN layer presents areas of greater surface regularity than those of the alumina, which has a much more abrupt surface. These results are coincident with those from microscopy techniques. Also, Fig. 7(b) proves that Al_2_O_3_ topology, as TEM and SEM micrographs indicate, presents holes along its entire surface, being probably one of the reasons, together with the Al_2_O_3_ roughness itself, for the non-single crystalline growth of the GaN. Still, as previously indicated, the results so far reveal a good progression in the direction of obtaining a single-crystalline GaN layer deposited on a ceramic substrate. The GaN deposition by itself causes a smoothering of the surface provided by the mesoporous Al_2_O_3_.

**Figure 8:**
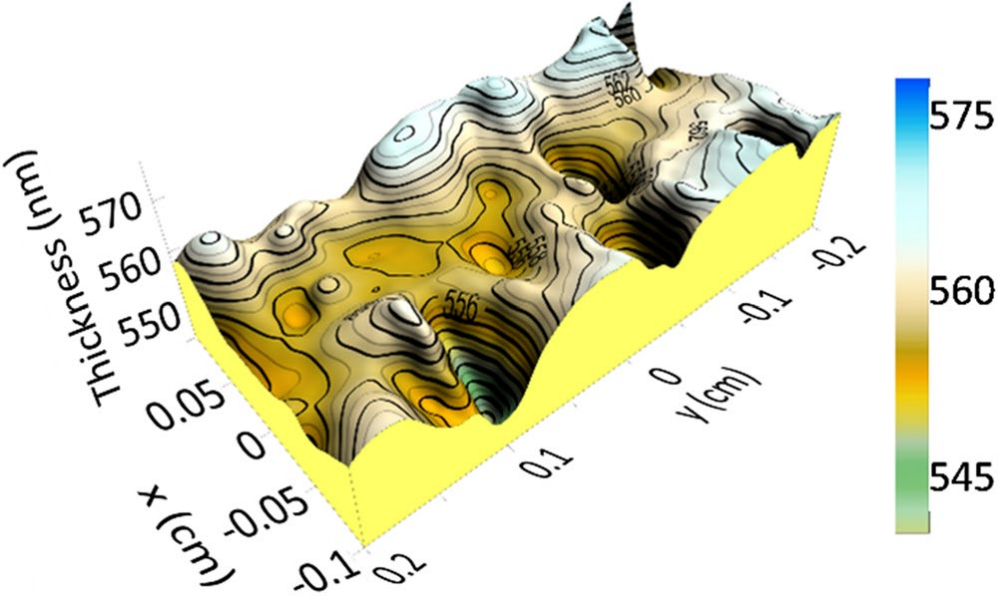
Topological map for GaN in sample SC1.

The same approach has been used for the topology study of one of the GaN/SiCer samples, SC1, but in this case an optical model consisting in a roughness/GaN/SiO_2_/Si was used. The thickness of the SiO_2_ layer has been fixed to 2 nm, according to TEM data. The thickness obtained for the GaN layer was (551.5 ± 0.8) nm in the measurement carried out with the 200 µm size probe with a roughness layer of (23.5 ± 0.4) nm and a thickness non-uniformity of only 2.6%. As in the case of CT4, the GaN layer thickness obtained by SE is in good agreement with measurements from TEM images (Table 1 and Fig. 4(f)). Figure 8 shows the SE topological map for the GaN layer for the same sample, also shown without the surface roughness layer, onto the SiCer substrate along a surface area of 2 × 4 mm^2^. The thickness variation across the measured area is only 5% of the layer thickness. Comparing the results achieved for LTCC and SiCer substrates it can be concluded that the growth of GaN on LTCC covered with Al_2_O_3_ shows a comparable surface roughness and layer homogeneity.

### Chemical composition and crystallographic nature

#### Chemical composition

TEM working in scanning mode (STEM) allows the application of a variety of chemical sensitive techniques with a high spatial resolution, such as High Angle Annular Dark Field (HAADF) imaging and Energy Dispersive X-Ray (EDX) spectroscopy. For this, it has to be taken into account that the intensity in HAADF images is proportional to *t·*Z^*n*^, where *t* represents the specimen thickness crossed by the electron beam and Z is the averaged atomic mass of the material that interacts with the electron beam, with *n* ranging between 1.6 and 1.9 in most cases^19^. Therefore, the contrast in these kind of images indicates a change in chemical composition (it can also indicate a change in the thickness of the sample in the direction to the electron beam propagation, but this issue is solved with adequate smooth TEM sample preparation processes, as those applied here). HAADF images have been obtained using a 1 nm STEM probe and an 8 cm camera length, which corresponds to a 20 mrad inner collecting angle for the HAADF detector. On the other hand, EDX spectra have been obtained in standard-less mode using the same STEM probe.

**Figure 9:**
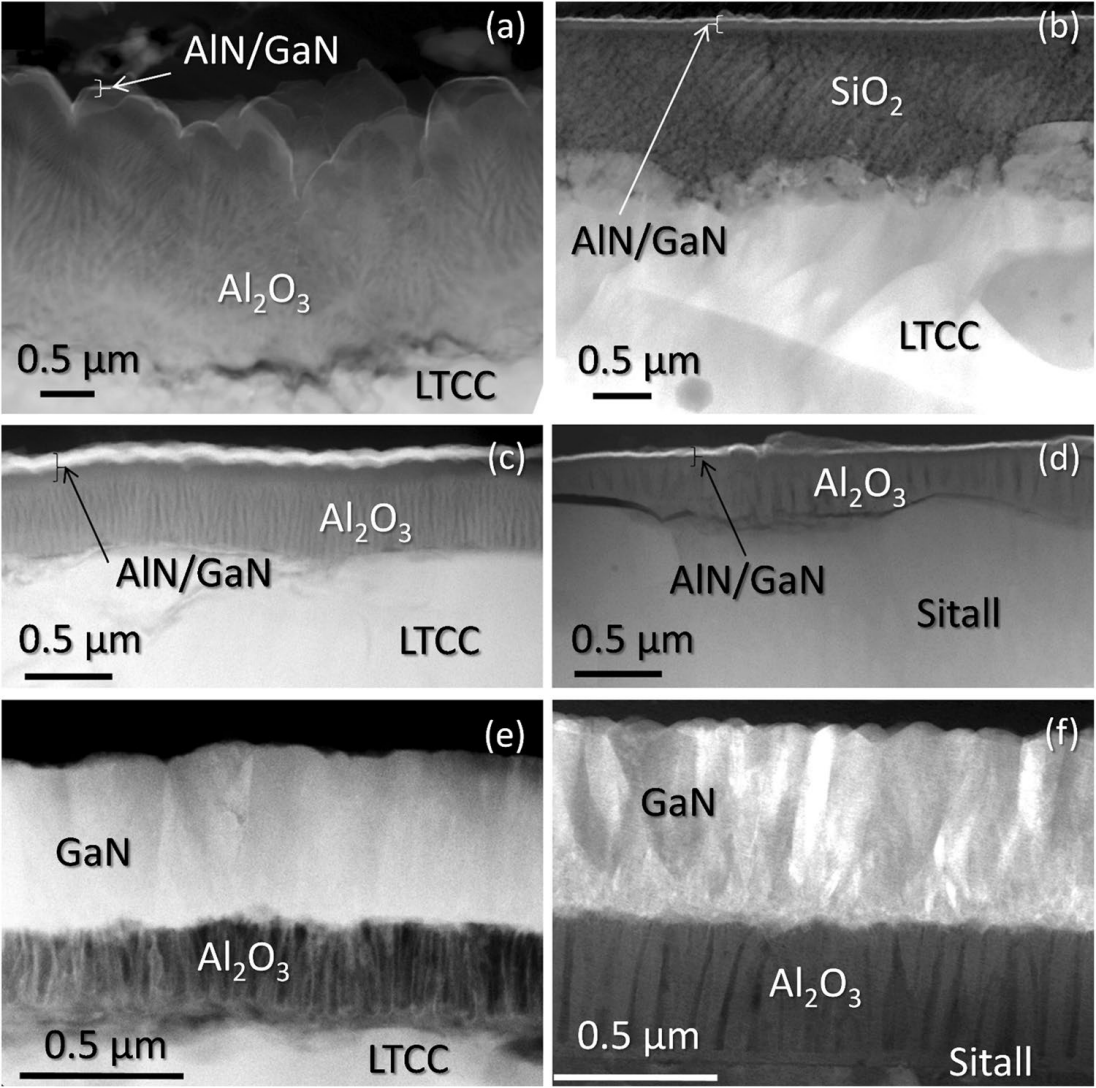
HAADF micrographs of heterostructures with Al_2_O_3_/LTCC and with other intermediate layers and substrates for the GaN: samples CT1. (**a**) CT2 (**b**) CT3 (c) ST1 (**d**) CT4 (**e**) and ST2 (**f**).

Figure 9 presents HAADF images of various heterostructures for different series. These images give an overview of the evolution of the GaN layer along the study, regarding its thickness and chemical composition. As it can be observed, a chemically continuous and homogeneous GaN layer has been achieved for all the heterostructures, and there are no compositional defects regardless of which material was utilized as substrate and transition structures for the nitride. On these HAADF images, dark elongated shapes are also observed in the Al_2_O_3_ layers. Such features, which correspond to locally thinner specimen regions, allow confirming the presence of elongated voids mentioned previously.

**Figure 10:**
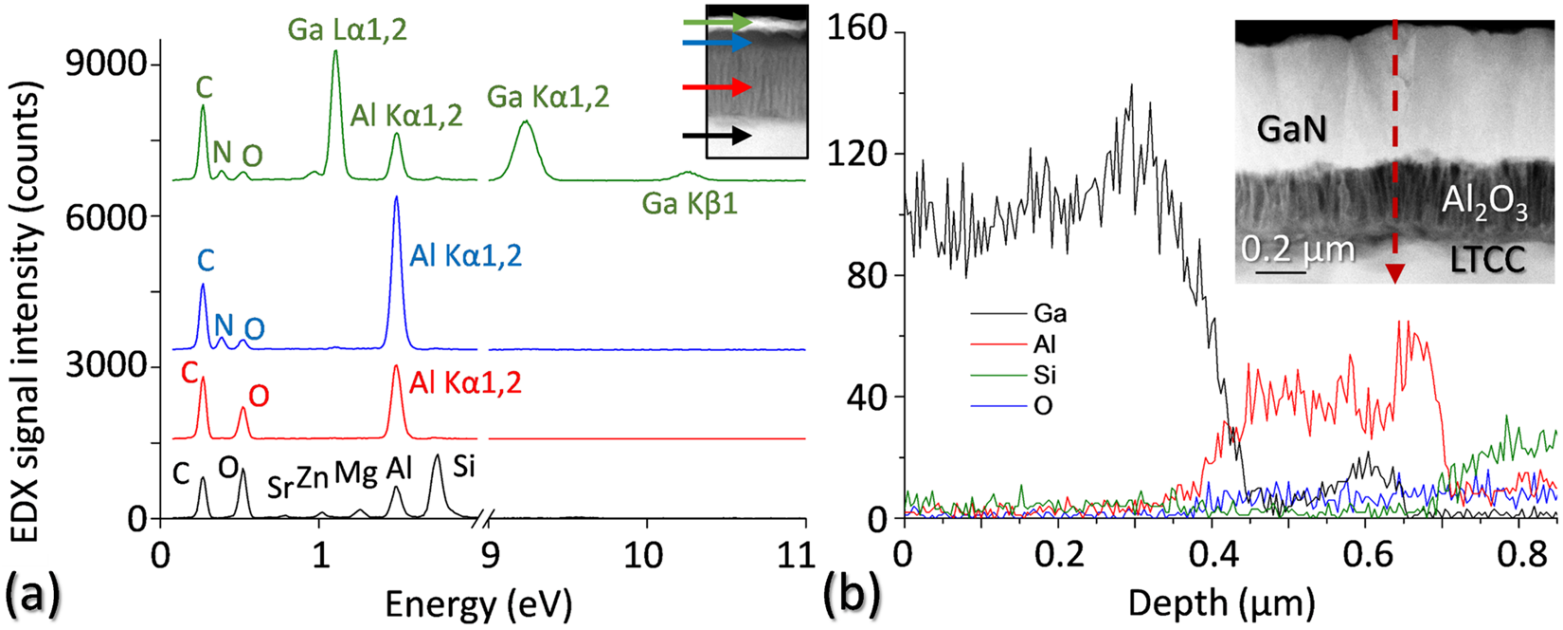
EDX punctual spectra taken at different layers (indicated arrows in the inset) in sample CT3 (**a**) and EDX linescan (trajectory shown by red discontinuous line in the inset) for sample CT4 (**b**).

These affirmations are also supported by compositional quantitative results. Punctual EDX spectra (Fig. 10(a)) corroborate that the top bright layers at Fig. 9 HAADF images are, indeed, gallium nitride. Note that the small aluminum signal in the EDX punctual spectrum for GaN layers is due to a projection of the AlN buffer layer, explainable regarding to the surface roughness. These techniques also confirmed the composition for those layers not common to most of the materials (Si substrate and SiO_2_ interlayer in SiCer and SiO_2_ sol gel in sample CT2), though the corresponding spectra are not shown here. Similarly, these types of spectra were taken for the thick top layer in specimens from the last series, revealing that it was also GaN. As a visual example, an EDX linescan for sample CT4 is presented in this figure, illustrating such affirmations. Again in this case, the presence of both Ga and Al signal at the GaN/Al_2_O_3_ interface may be due to surface roughness.

#### Crystallographic nature

In the long term, the achievements in this work are directed towards the deposition of monocrystalline GaN acting as active material for transistors for PE and high frequency devices. Therefore, it is imperative to characterize the crystallographic structure of the achieved nitride for each case.

**Figure 11:**
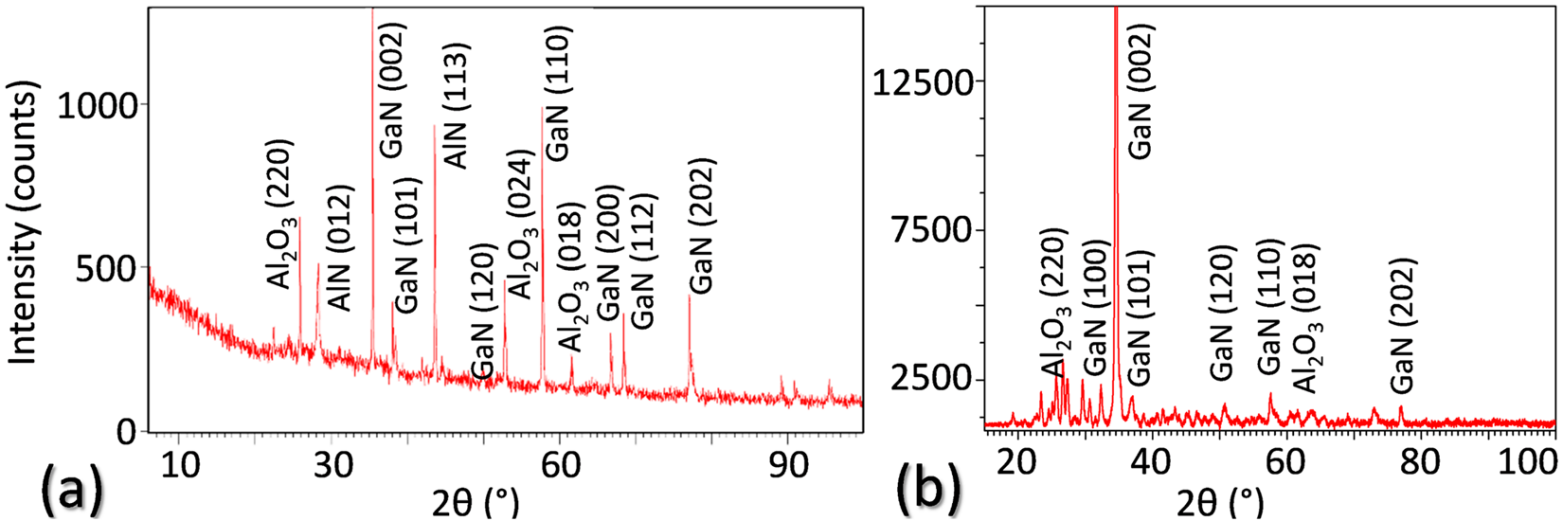
XRD theta-2theta curves acquired from the surface of specimens CT1 (**a**) and CT4 (**b**).

One first approach to this characterization is the use of XRD. The diffractograms in Fig. 11 reveal the achieved GaN evolution from a polycrystalline structure (Fig. 11(a), representative for those samples with GaN deposited using a RF-plasma source) to a mosaic structure with a clear preferential direction (exemplified by Fig. 11 (b)). The coincidence of the diffraction peaks with those for GaN atomic plane distances^20^ supports the EDX results about the composition of the top layer.

**Figure 12:**
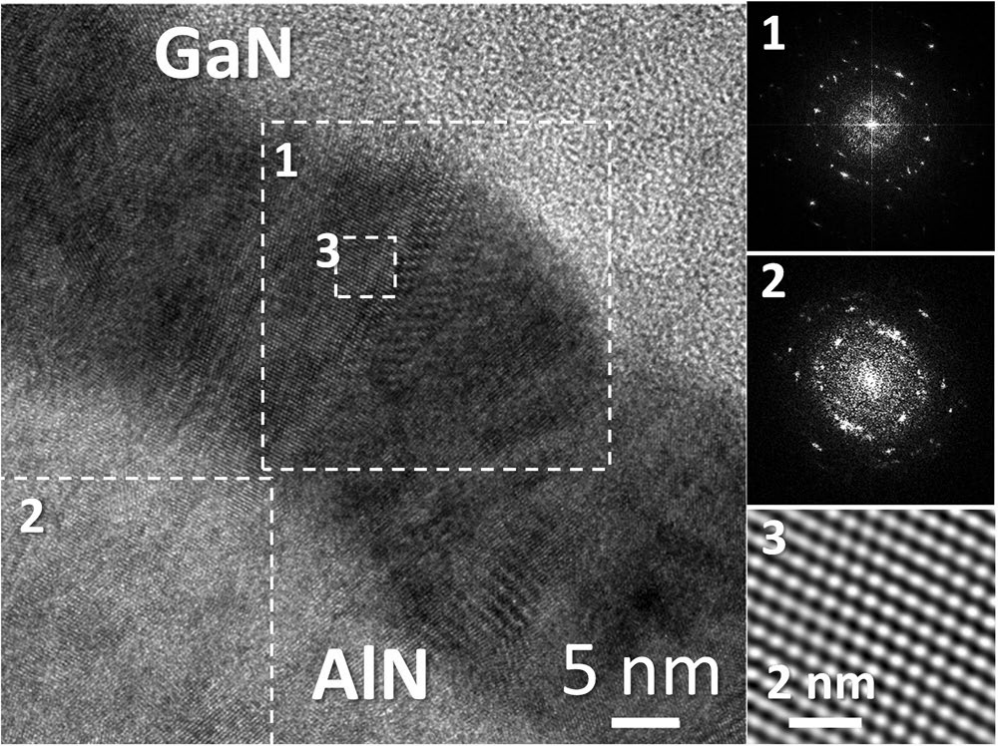
HRTEM micrograph of AlN/GaN for sample CT1. The dotted frames indicate the areas where the PS have been calculated and the bandpass filter has been applied.

A HRTEM micrograph for the AlN/GaN bilayer in sample CT1, representative of this structure in samples using RF-plasma for the III-N growth, is presented in Fig. 12. Power spectra are presented at the right side of the image, obtained from local areas indicated by dotted squares in the image. Note that a power spectrum (PS) is equivalent to an electron diffraction pattern with some limitations like (i) the resolution is governed by imaging condition, and (ii) the tilt cannot be estimated from a PS as it would be possible from a diffraction pattern. It can be concluded that GaN crystalline quality did not improved significantly while using RF-plasma, maintaining a polycrystalline nature, for both the GaN and the AlN layers, as revealed by the PS, with the characteristic ring-shape of such structures. Nevertheless, it was possible to find small monocrystalline areas, like the one presented in the third inset, at the right bottom corner of the figure. Most of those single crystalline regions revealed a cubic atomic structure, showing the characteristic ABCABC atomic ordering once the [011] direction is parallel to the incident electron beam. In order to allow a better observation of the detail, a bandpass filter has been applied to this part of the HRTEM image.

**Figure 13:**
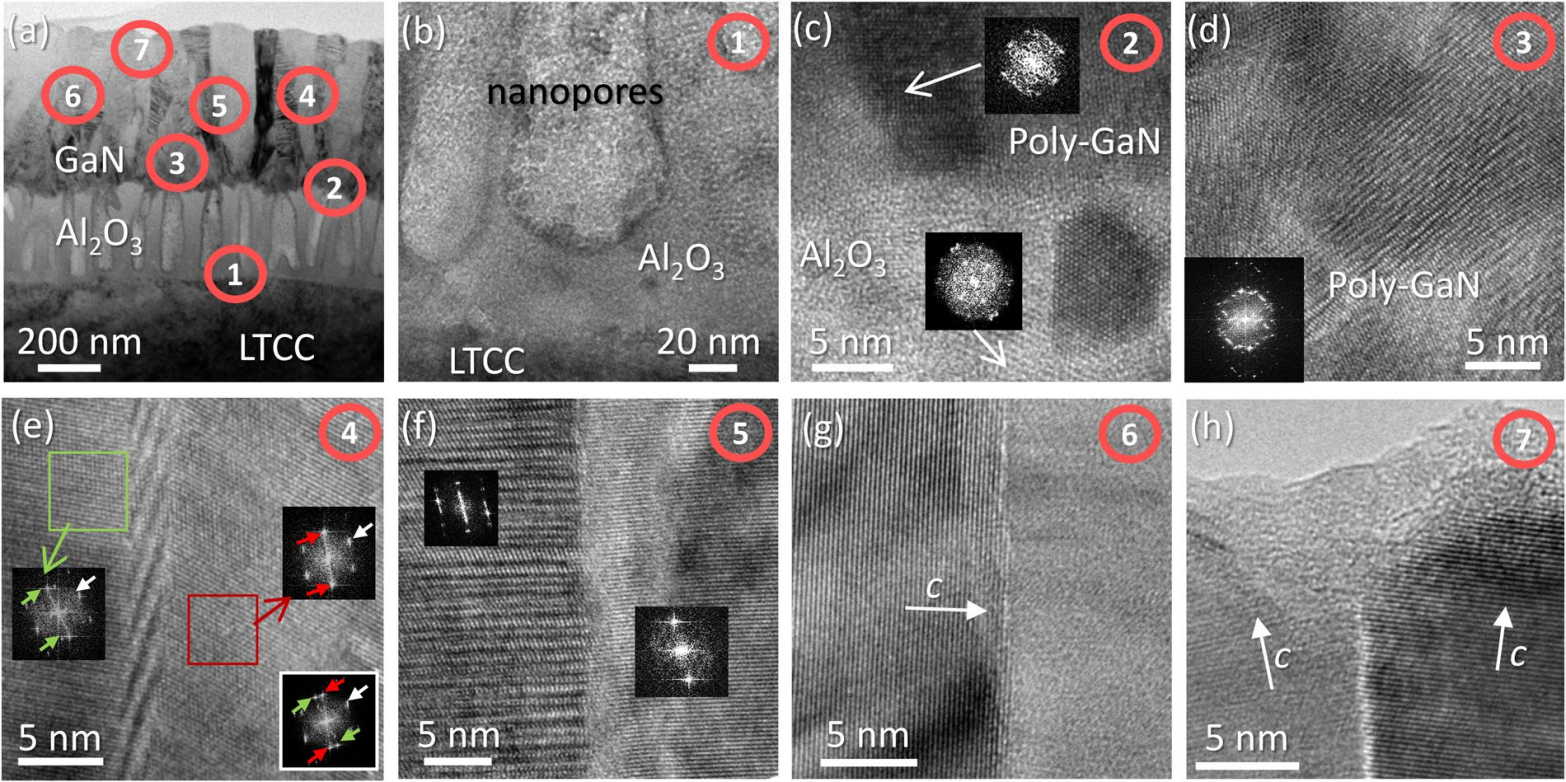
BF-CTEM image of sample CT4 (**a**) and HRTEM micrographs corresponding to the labeled positions in the BF-CTEM image (**b**–**h**).

**Figure 14:**
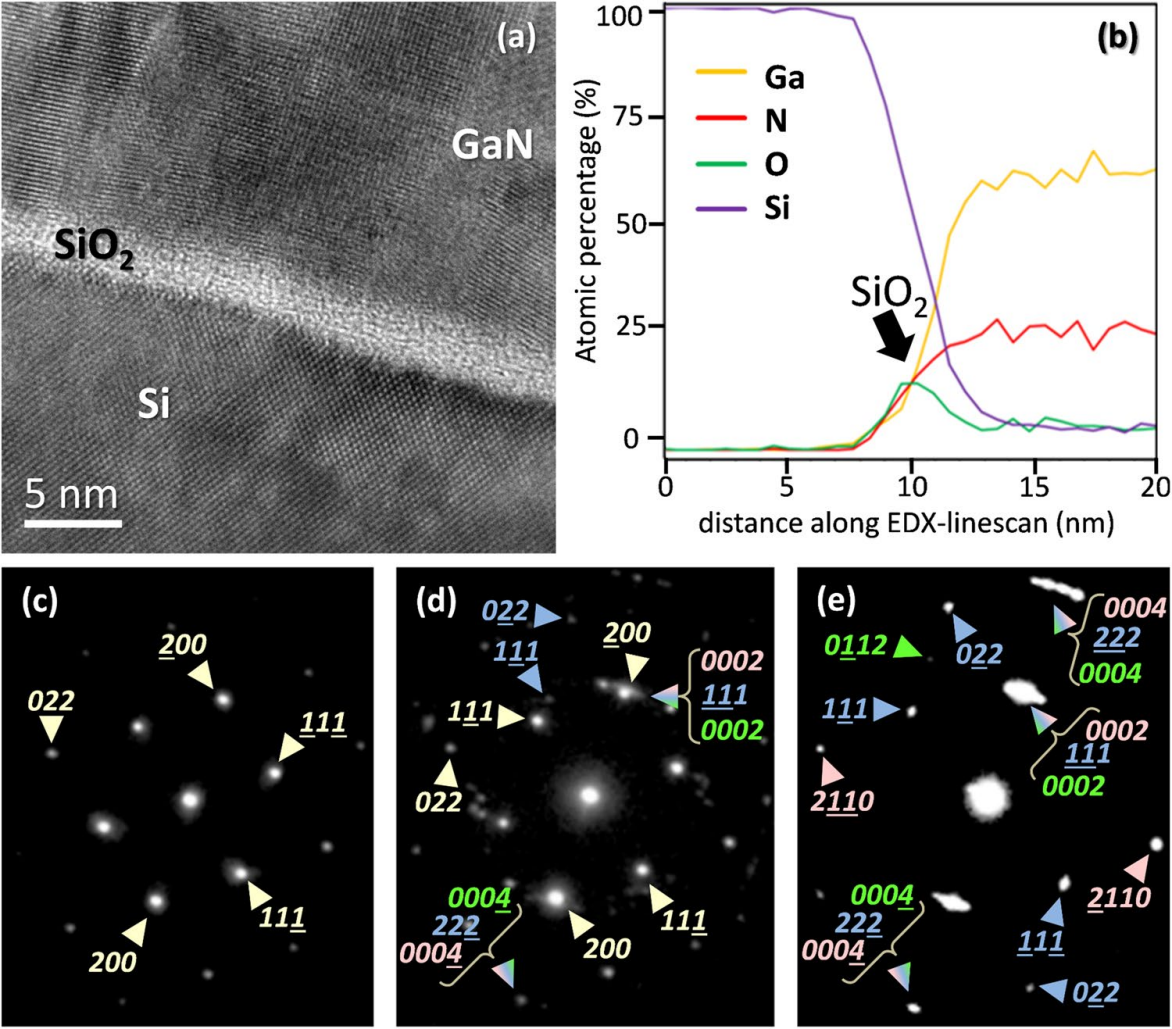
HRTEM micrograph. (**a**) EDX line scan (**b**) and SAED patterns (**c**)–(**e**) of sample SC2 in areas close to the Si/SiO_2_/GaN interphase. The color code for the reflections is: pale yellow and blue for cubic silicon and 3C-GaN close to their [011] zone axis, respectively; and pink and green for hexagonal 2H-GaN close to the [01$\bar{\text{1}}$ 0] and [2$\bar{\text{1}}\bar{\text{1}}$0] zone axis, respectively.

On the other hand, Figs 13 and 14 present a detailed study on the atomic arrangement of the GaN in specimens fabricated with a DC-plasma source and the largest growth time (CT4, ST2, SC1 and SC2), which gives a closer look at the steps of the GaN growth mechanism in those samples. The BF-CTEM micrograph in Fig. 13(a), similar to the one in Fig. 4(e), indicates the areas at where HRTEM images have been recorded and presented in the other images in this figure: LTCC/Al_2_O_3_ (Fig. 13(b)) and Al_2_O_3_/GaN (Fig. 13(c)) interfaces, and areas of GaN showing domain frontiers between multiple (Fig. 13(d)) or two (Fig. 13(e)–(h)) monocrystalline grains.

Figure 13(b) shows in more detail the nanopores crossing the Al_2_O_3_ and the abrupt well-adhered interphase between the substrate and intermediate layer in sample CT4, formed by the junction of the ceramic and the alumina likely by physical adsorption after sputtering, and later by chemical reaction during the post-growth applied heating. Despite its roughness, the Al_2_O_3_/GaN interphase (Fig. 13(c)) is continuous and chemically sharp. Thus, it can be concluded that both regions exhibit a high adherence. Also, it is possible to distinguish some crystalline formations in both phases in Fig. 13(c), which gives a good idea about how the nitride is deposited during the first steps of the Metal Organic Chemical Vapor Deposition (MOCVD) process. The GaN tries to accommodate to the alumina rough surface forming nanocrystalline seeds, which, in many places, is formed mainly by a cubic (zinc-blende) structure, as it can be deduced from the PS in the inset of the image, corresponding to the local area that is indicated by an arrow in that image. In any case, when the thickness of the GaN layer increases, this semiconductor behaves at first as a polycrystal (Fig. 13(d)), formed by many randomly oriented crystalline domains (see PS), which remains mainly cubic-structured. It is worth to mention that while the hexagonal structure is thermodynamically more stable for nitrides, it is not uncommon to find cubic regions in non-optimized materials^21^.

At higher layer thicknesses, these domains grow and coalescence, which lead to the formation of larger monocrystalline domains (Fig. 13(e)–(g)), thus, conditioning the posterior growth of the crystalline nitride columns, since their closed packed atomic planes are oriented perpendicular (or close) to the growth direction. In Fig. 13(e), a cubic structured twin-domain is presented. The PS of those domains (insets in the image, obtained from the areas framed by green and red squares in the image) show the [011] GaN zone axis, while the PS for the whole image (inset in the bottom right corner) reveals the transition between both structures. These monocrystals are tilted a certain angle among themselves, as can be observed throughout the 11 $\bar{\text{1}}$ reflection, or diffraction peak, corresponding to each one of them and which are indicated by red and green arrows in the PS. In spite of this change in crystallographic orientation, the cubic structures conserve the same vertical alignment, as indicated by the fact that the 200 reflection, pointed by a white arrow, does not change its position. Figure 13(f), on the other hand, shows the frontier between a wurtzite (hexagonal) GaN domain (left) and another one (right), in which only atomic fringes are visible due to the existence of a twist, not only a tilt, between both grains. The separation between both domains, as expected at the joint of two different crystallographic structures, presents multiple planar defects and stacking faults.

Figure 13(g) shows an atypical feature, since it can be observed that the (0002) atomic planes (single hexagonal GaN crystal at the left of the image) are perpendicular to the growth direction. The nitride in this domain, therefore, has grown in a semipolar direction, with a prismatic plane parallel to the layer surface. This, however, is not an extended phenomenon along the GaN layer of this series, in which a behavior such as the one presented in Fig. 13(h) is the most common: hexagonal structures slightly tilted, forming the nitride layer mosaic structure.

A similar behavior was observed when studying those samples fabricated using non-porous substrates and DC-plasma source (ST2, SC1 and SC2). Figure 14 gives a detailed view of the substrate/intermediate layer/GaN interphase. The HRTEM image (Fig. 14(a)) shows an amorphous thin interlayer on the Silicon crystal (it was tilted in many directions and contrasts associated to lattice fringes did not arise), and the nitride placed on top of it, grown as a crystalline layer. We suspect that this amorphous nanolayer is a rest of the Si native oxide that could not be completely removed, and that was amorphous or later amorphized during ion milling along the TEM preparation. Since it would be surprising for a crystalline (or close to it) structure to grow on top of an amorphous, this kind of explanation was given before for similar systems^22–24^. Also, an EDX linescan spectrum (taken along the interphase in Fig. 14(a), going from the silicon substrate to the GaN layer) is presented in Fig. 14(b). This linescan confirms that the amorphous layer in the HRTEM image is indeed SiO_2_.

The Selected Area Electron Diffraction (SAED) patterns in Fig. 14(c)–(e), obtained using 100-500 nm apertures and a camera length of 120 cm, illustrate the GaN similar atomic structure in these samples with that in sample CT4, observed in Fig. 13. Figure 14(c) corresponds to the diffraction pattern taken for an area inside the silicon layer, close to the amorphous interlayer. As expected, it shows the reflections close to the [011] zone axis belonging to the cubic system associated to silicon. The small thickness of the SiO_2_ interlayer allows to take a SAED including all the materials in this interphase, as presented in Fig. 14(d). This SAED shows strong diffraction peaks, corresponding to the planes of the material with the largest monocrystalline volume included in the region of interest (silicon), and also reflections that are associated to different monocrystals with nanometric size, which form a volume close to a polycrystal, but predominantly with cubic domains. Of course, due to the amorphous nature of the SiO_2_, no peaks related to this intermediate layer appear in this diffractogram. It can be observed that the reflections associated to the silicon {200} and the GaN {0002} and {1$\bar{\text{1}}\bar{\text{1}}$} atomic plane families are coincidental, since their lattice plane spacings are approximately the same, but there is an slight angle variation in each of those reflections (which, in the case of those peaks in the SAED in Fig. 14(e), due to the intensity saturation, is seen as an arched peak), indicating a tilted growth. Finally, two GaN polytypes (wurtzite and zinc-blende) with three different orientations are revealed in the diffraction pattern in Fig. 14 (e). In any case, these structures maintain their {0002} and {1$\bar{\text{1}}\bar{\text{1}}$} plane families almost parallel, which is an indication of the fact that those grains are producing a polar hexagonal GaN even in the beginning of the nitride deposition (differently to the case of specimen CT4), but with a tilt between the crystallographic structures of the adjacent grains, thus forming, in samples ST2, SC1 and SC2, the same mosaic arrangement for the GaN that is produced in sample CT4.

## Conclusions

GaN heterostructures have been fabricated by a low-temperature MOCVD technique using three sets of substrates and three different approaches regarding the growth temperature, its duration and the type of plasma source. Along this work, an evolution on the thickness and crystalline quality of the GaN-on-LTCC has been perceived. In those materials with unpolished LTCC substrate, nano-poly-crystalline layers of 15 nm of GaN on top of AlN buffer were formed. When used a polished substrate and with the same plasma source than the one in the previous case (RF-plasma), the GaN layer, with an abrupt separation to the AlN underneath, improved in terms of crystallinity, roughness and thickness (up to 70 nm). Remarkably, the improved GaN that was fabricated using a DC-plasma source presents a mosaic microstructure with layers thickness larger than 500 nm, formed by single-crystalline columnar grains randomly misoriented (tilted) among each other, but aligned in the polar direction, or close to it, with respect to the substrate surface. When first deposited, the GaN arranges in cubic fashion predominantly, but tends to form hexagonal structures afterwards.

Results on the general structure, composition, topology and crystallinity have been reported. To the best of our knowledge, this work presents the highest crystalline quality obtained to the date for GaN grown on top of porous LTCC materials.

## Methods

Three sets of specimens have been studied in this work, each one related to a different type of ceramic or glass-ceramic substrate that was used for the deposition of GaN on top of intermediate and buffer layers. These substrates are: (a) HERATAPE® CT 700 from Heraeus (CT 700), a LTCC substrate^25^; (b) Sitall, a glass-ceramic bulk material based on the Al_2_O_3_-SiO_2_-MgO-TiO_2_-CeO_2_-La_2_O_3_ system^26^; and (c) SiCer, a composite formed by a thin Si single-crystalline wafer, bonded to a CTE adapted LTCC green body during sintering^27^. These substrates were either bought from commercial suppliers or fabricated at the Technical University of Ilmenau (TU Ilmenau). The studied specimens are collected in Table 1. Regarding the finishing of these substrates, unpolished (“as fired”) CT 700 was used for the first four samples, while in all the other cases, the substrates used were polished. In this study, SiCer substrates utilized Si (111) and Si (001), which, though a polishing process was not applied in this case, due to the Si crystallinity, the surface could be considered similar to a polished one, in relation to the subsequent material growth. SiCer substrates were sintered using pressure assisted sintering at 3.2 kPa, 900 °C, for 25 min. The substrates CT 700 were sintered at 875 °C peak temperature for 30 minutes. A tube furnace with a fused silica tube is used for sintering. This particular LTCC (CT 700) was selected due to its CTE (6.7 ppm/K), close to the one of the alumina layer deposited on top of the ceramic.

The alumina interlayers were produced in the form of nanoporous Al_2_O_3_ at the facilities of the Technical University of Sofia by voltastatic oxidation (40 V) in 0.3 M oxalic acid solution at 15 °C. The samples were gradually immersed by their Al-face in the electrolyte at a rate from 5 to 10 µm/s to obtain a complete transformation of the previously sputtered aluminium layer into a nanoporous aluminium oxide^28^. As a source of direct current, a power supply (40 V/5 A), manufactured by Voltcraft® Germany, was utilized. The immersion velocity is defined by the substrate roughness (LTCC or Sitall) and the thickness of the initial aluminum layer. Also, in sample CT2, a SiO_2_ layer produced by sol gel technology is used instead of alumina, being fabricated by the researchers at TU Ilmenau^29^.

Nitride semiconducting layers were deposited at Lakehead University by plasma assisted, low temperature MOCVD related technique, using a custom-built reactor with three chambers: a loading lock, a UHV chamber and a residual gas analyzer chamber along with a plasma source for nitrogen species. In fact, the 2 nm-thick SiO_2_ intermediate layer utilized for the growth of GaN on SiCer forms by Si ambient oxidation during the chamber-to-chamber specimen transportation process.

Regarding the GaN growth, it has to be taken into account that different approaches were applied for the fabrication of this compound: RF nitrogen plasma at 550 °C (samples CT1 and CT2) and at 540 °C (specimens CT3 and ST1), was used with varying pressures and “Nitrogen to Metal-Organic” precursor ratios in order to obtain a 2D growth of GaN. In the mentioned cases, a first step is applied for creating an AlN buffer layer with growing parameters kept in ways in which 3D growths are achieved (initial expectation of amorphous AlN to be formed). On the other hand, for samples CT4, ST2, SC1 and SC2, a DC plasma at 550 °C, applied a period three times longer than the one used in the second series, was utilized to grow directly GaN (without AlN). More details on the LTCC substrate composition and MOCVD parameters for the three series are given elsewhere^30^. Therefore, though samples are classified in different groups, attending to the substrate, these three different growth processes have to be, then, also taken into account when comparing the results regarding the achieved GaN layers.

Following the fabrication of these materials, topographic, structural and compositional characterizations were carried out through the use of techniques related to XRD, AFM, Optical Microscopy and SEM, TEM, STEM microscopies. The equipment employed to apply these techniques consisted, respectively, in a Bruker D8 Advance X-Ray Diffractometer, an AFM Bruker Multimode Nanoscope IIIa operating in tapping mode, a DSX510 Olympus digital microscope, a Field-Effect ZEISS GeminiSEM 500 SEM, a FEI Tecnai F30 TEM (operated at 300 kV), a FEG-2010 Jeol and a FEI TALOS STEM microscopes (both working at 200 kV). In order to carry out (S)TEM characterization, samples were first prepared in cross-section XTEM disposition at UCA and thinned down until electron-transparency using traditional grinding-polishing methods and ion-milled with Ar^+^ ions using a Gatan model 691 Precision Ion Polishing System. Specific details on the experimental setup for the different (S)TEM related techniques are indicated in further sections of this work.

SE was applied at UCA to investigate the topography of macroscopic areas of GaN and intermediate layers. SE measurements were performed in the spectral range between 450 and 950 nm with an automatic rotating analyzer J. A. Woollam V-WASE (variable angle) ellipsometer equipped with an automatic retarder.

### Data availability

All data generated or analyzed during this study are included in this published article (and its Supplementary Information files).
